# Golgi α-mannosidases regulate cell surface *N*-glycan type and ectodomain shedding of the transmembrane protease corin

**DOI:** 10.1016/j.jbc.2023.105211

**Published:** 2023-09-01

**Authors:** Hao Wang, Yi-Shi Liu, Yingfei Peng, Wei Chen, Ningzheng Dong, Qingyu Wu, Baishen Pan, Beili Wang, Wei Guo

**Affiliations:** 1 Department of Laboratory Medicine, Zhongshan Hospital, Fudan University, Shanghai, China; 2 Key Laboratory of Carbohydrate Chemistry and Biotechnology, Ministry of Education, School of Biotechnology, Jiangnan University, Wuxi, China; 3 Cyrus Tang Hematology Center, Collaborative Innovation Center of Hematology, State Key Laboratory of Radiation Medicine and Prevention, Soochow University, Suzhou, China; 4 NHC Key Laboratory of Thrombosis and Hemostasis, Jiangsu Institute of Hematology, The First Affiliated Hospital of Soochow University, Suzhou, China; 5 Cancer Center, Shanghai Zhongshan Hospital, Fudan University, Shanghai, China; 6 Department of Laboratory Medicine, Xiamen Branch, Zhongshan Hospital, Fudan University, Shanghai, China; 7 Department of Laboratory Medicine, Wusong Branch, Zhongshan Hospital, Fudan University, Shanghai, China; 8 Branch of National Clinical Research Center for Laboratory Medicine, Shanghai, China

**Keywords:** corin, dysfunction, ectodomain shedding, mannosidase, *N*-glycan

## Abstract

Corin is a transmembrane protease that activates natriuretic peptides on the cell membrane. Reduced cell surface targeting or increased ectodomain shedding disrupts cell membrane homeostasis of corin, thereby impairing its cell surface expression and enzyme activity. *N*-glycans are essential in corin ectodomain shedding. Lack of *N*-glycans promotes corin ectodomain shedding in the juxtamembrane and frizzled-1 domains. The nascent *N*-glycans, transferred onto the polypeptide of corin, undergo multistep *N*-glycan processing in the endoplasmic reticulum and Golgi. It remains unclear how trimming by Golgi α-mannosidases, the critical *N*-glycan processing steps in *N*-glycan maturation, may regulate corin biosynthesis. In this study, we examined the effects of kifunensine and swainsonine, the inhibitors for α-mannosidases I and II, on corin expression and function. Western analysis of corin proteins in cell lysates and conditioned media from the inhibitor-treated corin-stable HEK293 cells and AC16 cells showed that both α-mannosidases I and II were required to maintain complex *N*-glycans on cell surface corin and protect corin from ectodomain shedding in the juxtamembrane and frizzled-1 domains. Cell viability analysis revealed that inhibition of α-mannosidase I or II sensitized cardiomyocytes to hydrogen peroxide-induced injury *via* regulating corin. Moreover, either one of the two coding genes was sufficient to perform Golgi α-mannosidase I trimming of *N*-glycans on corin. Similarly, this sufficiency was observed in Golgi α-mannosidase II-coding genes. Inhibition of ectodomain shedding restored corin zymogen activation from kifunensine- or swainsonine-induced reduction. Together, our results show the important roles of Golgi α-mannosidases in maintaining cell membrane homeostasis and biological activities of corin.

Corin is a type II transmembrane serine protease mainly expressed in the heart (1). By activating atrial natriuretic peptide (ANP), the cardiac corin promotes sodium excretion and vessel relaxation in an endocrine mechanism (2, 3, 4). Corin is also expressed in the uterus (1, 5). During pregnancy, uterine corin activates ANP to promote trophoblast invasion and spiral artery remodeling (6, 7). In cells, corin is synthesized as a zymogen that is activated on the cell surface by proprotein convertase subtilisin/kexin 6 (8). Proprotein convertase subtilisin/kexin 6 is secreted in an intracellular trafficking pathway distinct from that of corin (Figs. 1*A* and S1). The cleaved corin protease domain is connected to the prodomain on the cell membrane *via* a disulfide bond (Figs. 1*A* and S1) (8). The cell surface corin is shed by a disintegrin and metalloproteinase (ADAM) 10 in the juxtamembrane domain or autocleaved at Arg-164 and Arg-427 in the frizzled-1 and low-density lipoprotein receptor-5 domains, generating three fragments of ∼962 AA (by ADAM10), and 878 and 615 AA (by the autocleavage), respectively (Figs. 1 and S1) (9). Reduced cell surface targeting or increased ectodomain shedding impairs corin cell surface expression and enzyme activity (10, 11). Human corin is predicted to have 19 *N*-glycosylation sites in its extracellular region (12, 13). Abolishing *N*-glycosylation impairs corin cell surface expression and zymogen activation (13, 14). Further studies have shown that *N*-glycosylation at different sites has distinct roles in corin biosynthesis. *N*-glycosylation at Asn-80 and Asn-231, for example, inhibits corin shedding in the juxtamembrane domain and the frizzled-1 domain, respectively (12). *N*-glycosylation at Asn-697 in the scavenger receptor domain and at Asn-1022 in the protease domain is important for corin cell surface targeting (12).

**Figure 1:**
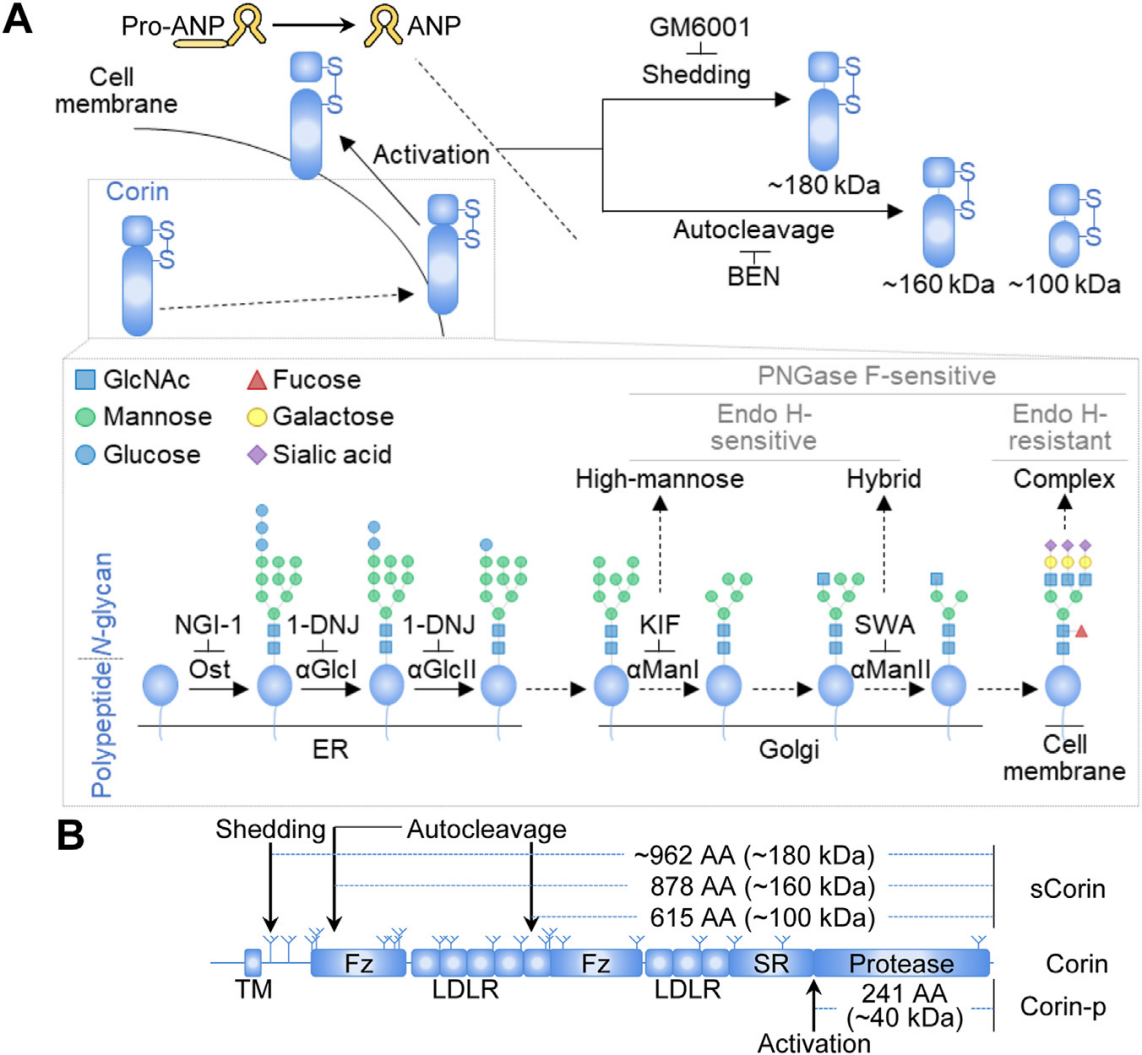
**Polypeptide and *N*-glycan processing of corin.***A*, corin biosynthesis and *N*-glycosylation. Corin is synthesized as a zymogen and activated on the cell surface. The activated cell surface corin converts pro-ANP to ANP. Cell surface corin is also shed (inhibitor: GM6001) or autocleaved (inhibitor: BEN), generating three soluble fragments. During the intracellular trafficking, *N*-glycans are transferred onto the polypeptide of corin by oligosaccharyltransferase (Ost) and processed by glycosidases and glycosyltransferases in the ER and Golgi. *N*-glycosylation inhibitors used include NGI-1 (for Ost), 1-DNJ (for α-glucosidase I, αGlcI, and α-glucosidase II, αGlcII), KIF (for α-mannosidase I, αManI), and SWA (for α-mannosidase II, αManII). Fully processed *N*-glycans are expected to be the complex type, with both branches containing monosaccharides other than mannose, and resistant to Endo H digestion. Dysfunction of *N*-glycan processing may disrupt the transfer of monosaccharides to one (hybrid type) or both (high-mannose type) branches, making *N*-glycans sensitive to Endo H digestion. *B*, human corin domains and *N*-glycosylation sites. The transmembrane (TM), frizzled (Fz), LDL receptor (LDLR), scavenger receptor (SR), and serine protease (protease) domains are shown. *N*-glycosylation, juxtamembrane shedding, and autocleavage sites are indicated. The corresponding corin bands on Western blots are indicated. ANP, atrial natriuretic peptide; BEN, benzamidine; NGI-1, *N*-linked glycosylation inhibitor-1; 1-DNJ, 1-deoxynojirimycin; KIF, kifunensine; SWA, swainsonine.

*N*-glycosylation is a multistep posttranslational modification (15, 16, 17, 18, 19). The two-branch 14-monosaccharide *N*-glycan core (Glc3Man9GlcNAc2; Glc, glucose; Man, mannose) is transferred onto nascent proteins by oligosaccharyltransferase in the endoplasmic reticulum (ER), which is the initial step of *N*-glycosylation (15, 17, 20). The following *N*-glycan processing in the ER and Golgi includes glycosidase trimming of glucose and mannose, and glycosyltransferase transfer of other monosaccharides (15, 17). Based on the sugar residues in the *N*-glycans, there are three main types of *N*-glycans: high-mannose, hybrid, and complex *N*-glycans (Fig. 1*A*). Considering that glycosidase trimming is prerequisite of the glycosyltransferase transfer and conversion from high-mannose to hybrid and complex *N*-glycans, elucidation of the effects of glycosidase trimming on protein expression and function is of biological importance. It has been found that glucosidase trimming is required for calnexin-assisted protein folding of human corin (18). However, the importance of mannosidase trimming in corin biosynthesis remains unknown, especially the trimming by Golgi α-mannosidases I and II, which determines the type of *N*-glycans on cell surface corin (Fig. 1*A*).

Pathophysiological factors or drugs may cause dysfunction of glycosidase trimming (16, 21, 22, 23, 24, 25). A homozygous truncation variant in *MAN2A2*, one of the two genes encoding Golgi α-mannosidase II, decreases MAN2A2 protein level and complex *N*-glycans in lymphoblasts (21). 1-deoxynojirimycin (1-DNJ, Duvoglustat), an agent with antihyperglycemic, antiobesity, and antiviral activities, is a potent inhibitor for α-glucosidase (both I and II) trimming (24, 25). Swainsonine (SWA) is an α-mannosidase II inhibitor, which reduces 5-fluorouracil tolerance and enhances the immune response to anti-programmed death ligand 1 in tumors (16, 22, 23, 26). Moreover, dysfunction of glycosidase trimming may contribute to dysregulation of protein and cellular functions. Inhibition of α-glucosidase trimming by 1-DNJ impairs corin cell surface expression and prothrombin secretion (18). Both SWA and 1-deoxymannojirimycin (α-mannosidase I inhibitor) decrease cell surface thyroid peroxidase activity, hence preventing iodide organification in cultured porcine thyroid cells (27).

In this study, we tested the hypothesis that Golgi α-mannosidases may regulate cell surface *N*-glycan type and biosynthesis of corin. We treated corin-expressing HEK293 and AC16 cells with kifunensine (KIF) and SWA, α-mannosidase I and II inhibitors that could retain *N*-glycans of high-mannose and hybrid types, respectively. Corin zymogen activation, ectodomain shedding, pro-ANP processing, and cell surface expression in the inhibitor-treated and/or gene KO cells were analyzed by Western blotting, immunoprecipitation, and flow cytometry. The *N*-glycan types on cell surface corin were verified by glycosidase digestion. Our results indicate that both Golgi α-mannosidases I and II are required to maintain complex *N*-glycans on cell surface corin and to prevent corin ectodomain shedding.

## Results

### Corin activation with N-glycosylation inhibitors

**Figure 2:**
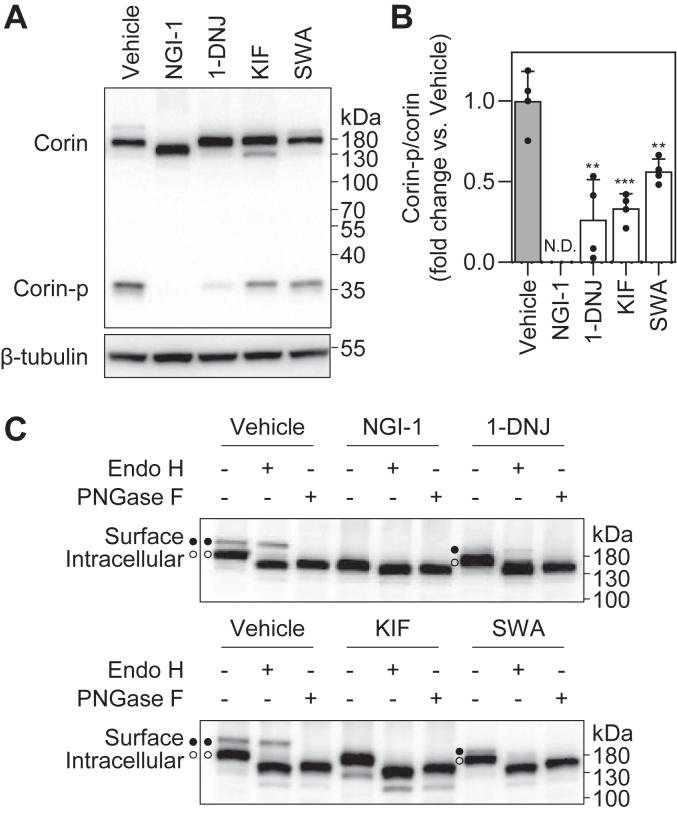
**Zymogen activation of corin with inhibitors of *N*-glycosylation.***A*, Western blot analysis of corin proteins in cell lysates from corin-expressing HEK293 cells treated with *N*-glycosylation inhibitors, including *N*-linked glycosylation inhibitor-1 (NGI-1), 1-deoxynojirimycin (1-DNJ), kifunensine (KIF), and swainsonine (SWA). The activation-cleaved corin protease domain (corin-p) band is shown. Levels of β-tubulin in cell lysates were used to assess protein amounts in each sample. *B*, ratio of corin-p *versus* corin bands, as estimated by densitometric analysis of Western blots. Data are means ± SD from four independent experiments. ∗∗*p* < 0.01; ∗∗∗*p* < 0.001 *versus* vehicle. *C*, Western blot analysis of corin in cell lysates with (+) or without (−) Endo H or PNGase F digestion. The cell surface (*black dots*) and intracellular (*white dots*) corin bands are indicated. ND, not detected; PNGase, peptide-*N*-glycosidase.

Human corin is synthesized as a zymogen that is subsequently activated on the cell surface (Figs. 1 and S1). The protease domain of the activated corin remains membrane-bound *via* a disulfide bond (Fig. 1*A*). During the intracellular trafficking, corin undergoes *N*-glycosylation and *N*-glycan processing (Fig. 1). To examine the importance of *N*-glycan processing, especially by α-mannosidases, in corin biosynthesis, we treated stable corin-expressing HEK293 cells with *N*-glycosylation inhibitors (Fig. 2*A*). In cell lysates from these cells, the corin proteins were expressed at similar levels, as indicated by comparable levels of the top zymogen bands (corin) on Western blots (Fig. 2*A*). Under the reducing conditions with DTT in Western blotting, the protease domain band (corin-p) of ∼40 kDa from the activated corin was detected in all the samples, except the *N*-linked glycosylation inhibitor-1 (NGI-1)-treated sample (Fig. 2*A*). Because corin is activated on the cell surface, the corin-p band also serves as an indicator of corin cell surface expression (8). The levels of corin-p bands were lower in 1-DNJ-, KIF- and SWA-treated samples than that in the vehicle-treated sample (Fig. 2*A*). The ratio of corin-p *versus* zymogen bands in these samples decreased to 26 ± 24, 33 ± 9, and 56 ± 8% of the vehicle-treated sample, respectively (n = 4; *p* = 0.003, 1-DNJ *versus* vehicle; *p* < 0.001, KIF *versus* vehicle; and *p* = 0.004, SWA *versus* vehicle) (Fig. 2*B*). These results indicate that, besides *N*-glycosylation and α-glucosidase trimming, α-mannosidase I and II trimming is also important for corin cell surface expression and zymogen activation.

As reported previously, the top corin zymogen band was removable by trypsin digestion of intact cells, indicating that this band represented the cell surface corin (18). The *N*-glycans on the cell surface corin appeared fully processed and mostly of the complex type, and thus resistant to endoglycosidase (Endo) H digestion, whereas the intracellular corin was mainly located in the ER with nascent *N*-glycans, which were Endo H-sensitive (Figs. 1 and 2*C*, vehicle). With NGI-1 treatment, neither Endo H nor peptide-*N*-glycosidase (PNGase) F digestion increased the migration of the intracellular corin band, which was at a similar position to that of the PNGase F–treated vehicle sample, indicating that no detectable *N*-glycans were added onto corin in the presence of NGI-1 (Fig. 2*C*). Interestingly, with 1-DNJ treatment, *N*-glycans on the cell surface corin remained Endo H-resistant (Fig. 2*C*). With SWA or KIF treatment, however, the cell surface corin zymogen band migrated faster and became indistinguishable from the intracellular corin band (Fig. 2*C*). In addition, the cell surface zymogen band from the SWA-treated sample was sensitive to Endo H (Fig. 2*C*). These results suggest that inhibition of α-glucosidase trimming by 1-DNJ did not prevent the formation of complex *N*-glycans on corin. In contrast, inhibition of α-mannosidases reduced the molecular mass of corin but did not block the cell surface expression of corin with high-mannose or hybrid-type *N*-glycans.

### Corin shedding with N-glycosylation inhibitors

**Figure 3:**
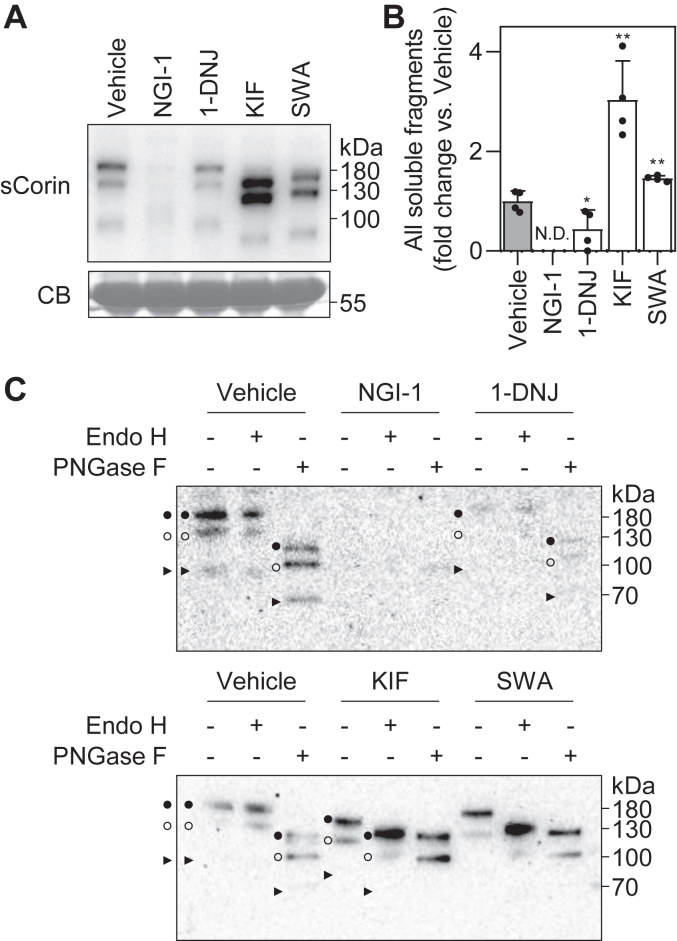
**Ectodomain shedding of corin with inhibitors of *N*-glycosylation.***A*, Western blot analysis of soluble corin (sCorin) in the conditioned medium from corin-expressing HEK293 cells treated with different *N*-glycosylation inhibitors. Levels of a Coomassie Blue (CB)-stained nonspecific protein in the conditioned medium were used to assess protein amounts in each sample. *B*, relative levels of all soluble fragments. Data are means ± SD from four independent experiments. ∗*p* < 0.05; ∗∗*p* < 0.01 *versus* vehicle. *C*, Western blot analysis of soluble corin in conditioned medium with (+) or without (−) Endo H or PNGase F digestion. The bands of the ∼962-AA (*black dots*), 878-AA (*white dots*), and 615-AA (*black arrowheads*) soluble fragments are indicated. ND, not detected; PNGase, peptide-*N*-glycosidase.

Ectodomain shedding is an important mechanism in regulating corin activity on the cell surface (11, 12). We next examined corin fragments in the conditioned media, instead of cell lysates, from the *N*-glycosylation inhibitor–treated HEK293 cells. In Western blotting under nonreducing conditions, three distinct bands at ∼180, ∼160, and ∼100 kDa, respectively, were detected in the vehicle-treated sample (Fig. 3*A*). These bands were hardly detectable in NGI-1–treated samples, whereas the levels of these bands decreased in 1-DNJ–treated samples (44 ± 39% of the vehicle, n = 4, *p* = 0.043) (Fig. 3, *A* and *B*). In contrast, the levels of the bands, especially the ones of ∼962 and 878 AA, increased in KIF- and SWA-treated samples (303 ± 78% of the vehicle for KIF, *p* = 0.002; 146 ± 5% of the vehicle for SWA, *p* = 0.005; n = 4) (Fig. 3*A* and *B*). These results indicate that inhibition of α-mannosidases may promote corin ectodomain shedding, whereas inhibition of oligosaccharyltransferase or α-glucosidases may prevent corin cell surface expression and ectodomain shedding.

Similar to the findings with cell surface corin bands in lysates, corin fragments from the conditioned medium migrated faster in KIF- and SWA-treated samples and were Endo H-sensitive (Fig. 3*C*). In contrast, 1-DNJ treatment did not alter the Endo H sensitivity of the soluble corin fragments from the conditioned media (Fig. 3*C*), as well as cell surface corin (Fig. 2*C*). The result is consistent with previous reports that inhibition of α-glucosidases did not affect subsequent *N*-glycan processing by α-mannosidases (19).

### Corin expression in AC16 cells treated with N-glycosylation inhibitors

**Figure 4:**
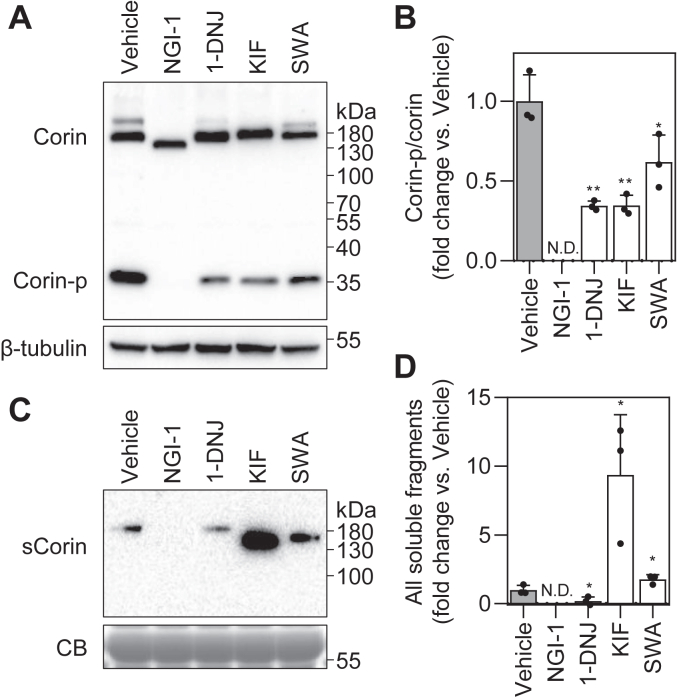
**Zymogen activation and ectodomain shedding of corin in AC16 cardiomyocytes treated with inhibitors of *N*-glycosylation.***A*, Western blot analysis of corin zymogen activation in the cell lysate from stably transfected AC16 cells expressing corin. Levels of β-tubulin in cell lysates were used to assess protein amounts in each sample. *B*, ratios of corin-p *versus* corin bands, as estimated by densitometric analysis of Western blots. *C*, Western blot analysis of corin ectodomain shedding in the conditioned medium from corin-expressing AC16 cells. Levels of a Coomassie Blue (CB)-stained nonspecific protein in the conditioned medium were used to assess protein amounts in each sample. *D*, relative levels of soluble fragments in samples. Data are means ± SD from three independent experiments. ∗*p* < 0.05; ∗∗*p* < 0.01 *versus* vehicle. ND, not detected.

Corin is mainly expressed in the heart (1, 8). We also examined the effects of *N*-glycosylation inhibitors on corin in AC16 cells, a human cardiomyocyte–derived cell line (28). Corin fragments in cell lysates and the conditioned media were examined by Western blotting (Figs. 4 and S2). In cell lysates, the ratio of corin-p *versus* zymogen bands in 1-DNJ-, KIF-, and SWA-treated samples decreased to 35 ± 3, 35 ± 6, and 62 ± 17% of the vehicle, respectively (n = 3; *p* = 0.003, 1-DNJ *versus* the vehicle; *p* = 0.003, KIF *versus* the vehicle; *p* = 0.049, SWA *versus* the vehicle) (Fig. 4, *A* and *B*). The results were similar to those observed in HEK293 cell–derived lysates.

Unlike in the conditioned medium from HEK293 cells, only the ∼962-AA corin fragment was detected in the conditioned medium from AC16 cells (Fig. 4*C*). This band was undetectable in the NGI-1–treated sample (Fig. 4, *C* and *D*). Levels of this band decreased in 1-DNJ–treated sample (20 ± 30% of the vehicle for 1-DNJ, *p* = 0.036; n = 3) and increased in KIF- and SWA-treated samples (937 ± 438% of the vehicle for KIF, *p* = 0.030; 177 ± 33% of the vehicle for SWA, *p* = 0.047; n = 3) (Fig. 4, *C* and *D*). In glycosidase digestion assays, the cell surface corin and the soluble fragment from AC16 cells behaved similarly to those from HEK293 cells (Fig. S2). These results indicate that the ∼962-AA band probably is the predominant shedding fragment in AC16 cells and that the effects of *N*-glycosylation inhibitors on corin biosynthesis are similar in HEK293 and AC16 cells.

### Reduced cell surface expression of corin with N-glycosylation inhibitors

**Figure 5:**
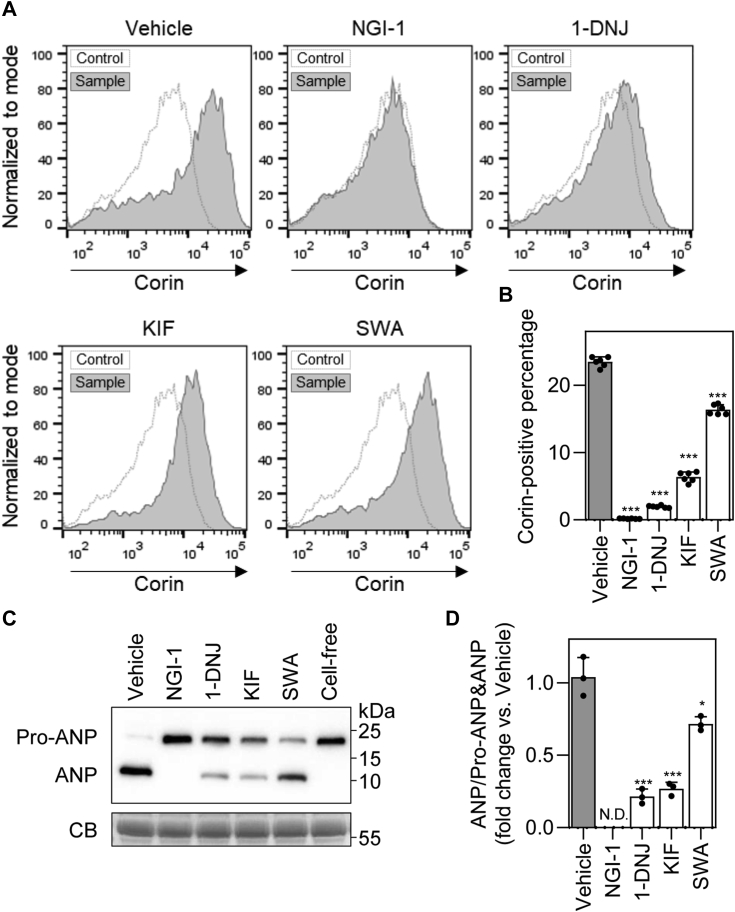
**Cell surface corin expression and pro-ANP processing activity.***A*, flow cytometric analysis of cell surface corin in corin-expressing HEK293 cells–treated with *N*-glycosylation inhibitors. The cells incubated with mouse IgG as a primary antibody were used as a control in the flow cytometry. Data are representative of each sample group. *B*, percentages of corin-positive cells in flow cytometry. Data are means ± SD from six independent experiments. *C*, Western blot analysis of pro-ANP processing by *N*-glycosylation inhibitor-treated corin-expressing HEK293 cells. The conditioned medium containing human pro-ANP was incubated with the inhibitor-treated cells or without cells (cell-free). Pro-ANP and ANP fragments in the conditioned medium were analyzed by immunoprecipitation and Western blotting. Levels of a Coomassie Blue (CB)-stained nonspecific protein in the conditioned medium were used to assess protein amounts in each sample. *D*, relative pro-ANP processing activities of the inhibitor-treated cells, as estimated by densitometric analysis of Western blots. Data are means ± SD from three independent experiments. ∗*p* < 0.05; ∗∗∗*p* < 0.001 *versus* vehicle. ND, not detected; ANP, atrial natriuretic peptide.

Corin zymogen activation occurs on the cell surface. To quantify corin expression on the cell surface, flow cytometry was performed with intact stable corin–expressing HEK293 cells. Percentages of corin-positive cells were lower in NGI-1-, 1-DNJ-, KIF-, and SWA-treated cells compared with that in the vehicle control (0.2 ± 0.0, 2.0 ± 0.2, 6.4 ± 0.7, and 16.4 ± 0.7%, respectively, *versus* 23.5 ± 0.7% in the vehicle; n = 6; all *p* values < 0.001) (Fig. 5, *A* and *B*). These results show that corin protein levels on the cell surface were reduced in the inhibitor-treated HEK293 cells, consistent with the observed effects of *N*-glycosylation inhibitors on corin zymogen activation.

### Pro-ANP processing assay

Corin activity is critical for pro-ANP processing. We investigated if *N*-glycosylation inhibitors affect the conversion of pro-ANP to ANP. We incubated recombinant human pro-ANP with stable corin-expressing cells without or with the *N*-glycosylation inhibitors. In the sample without corin-expressing cells (cell-free), no pro-ANP to ANP conversion was detected, while the conversion was detected, as indicated by the ANP band, in the samples incubated with vehicle-treated corin-expressing cells (Fig. 5*C*). In the NGI-1–treated sample, which had little corin zymogen activation, ANP band was undetectable (Fig. 5*C*). In 1-DNJ-, KIF-, and SWA-treated samples, which had reduced corin zymogen activation, ANP bands were weaker than that in the vehicle-treated sample (Fig. 5*C*). In quantitative analysis of pro-ANP and ANP bands, reduced pro-ANP to ANP conversion was confirmed in the cells treated with 1-DNJ (21 ± 5% of the vehicle; n = 3; *p* < 0.001), KIF (27 ± 4% of the vehicle; n = 3; *p* < 0.001), and SWA (72 ± 5% of the vehicle; n = 3; *p* = 0.018) (Fig. 5*D*). These results are consistent with the observed effects of *N*-glycosylation inhibitors on corin zymogen activation and cell surface expression.

### N-glycans in 3D models of corin domains

**Figure 6:**
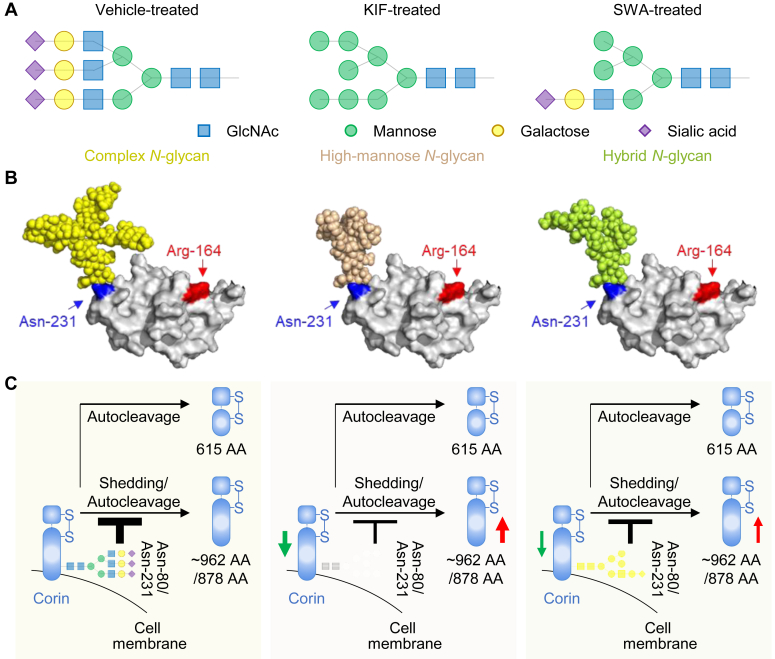
**3D models of corin domains with *N*-glycans.***A*, representative *N*-glycan structures with inhibition of Golgi α-mannosidases. *B*, surface models of corin frizzled-1 domains with different types of *N*-glycans. Complex (*yellow*), high-mannose (*wheat*), and hybrid (*limon*) *N*-glycans are displayed in the *sphere mode*. The *N*-glycosylation (Asn-231, *blue*) and autocleavage (Arg-164, *red*) sites are indicated. *C*, schematic diagram showing how inhibition of Golgi α-mannosidases may alter transmembrane protease corin activity *via N*-glycans. *N*-glycans at Asn-80/Asn-231 may block the access of shedding/autocleavage enzymes for generating ∼962/878-AA soluble fragments. KIF/SWA-caused reduction of *N*-glycan sizes at Asn-80/Asn-231 may increase ∼962/878-AA soluble fragments, thereby decreasing cell surface corin. KIF, kifunensine; SWA, swainsonine.

Increased ectodomain shedding might result in reduced corin cell surface expression and enzyme activity. *N*-glycans, at Asn-80 and Asn-231, have been shown to inhibit corin ectodomain shedding (10, 11, 12). To understand the potential effect of altered *N*-glycan types by Golgi α-mannosidase inhibition on corin ectodomain shedding, 3D models of corin domains were simulated with representative *N*-glycan structures produced under the inhibition of Golgi α mannosidases (Fig. 6*A*). As mentioned above, high-mannose *N*-glycans were produced with KIF treatment, while hybrid *N*-glycans were produced with SWA treatment (Figs. 1*A* and 6*A*). With the addition of galactoses and sialic acids onto either branch, the resulting *N*-glycans occupied more space, which is more likely to block the access of the proteolytic enzymes to the cleavage sites (juxtamembrane region for shedding and Arg-164 for autocleavage) (Figs. 6*B* and S3). These results indicate that KIF/SWA-caused reduction of *N*-glycan sizes at Asn-80/Asn-231 may increase ectodomain shedding of ∼962/878-AA fragments, thereby decreasing cell surface corin (Fig. 6*C*).

### Biological effects of Golgi α-mannosidase–regulated ectodomain shedding

**Figure 7:**
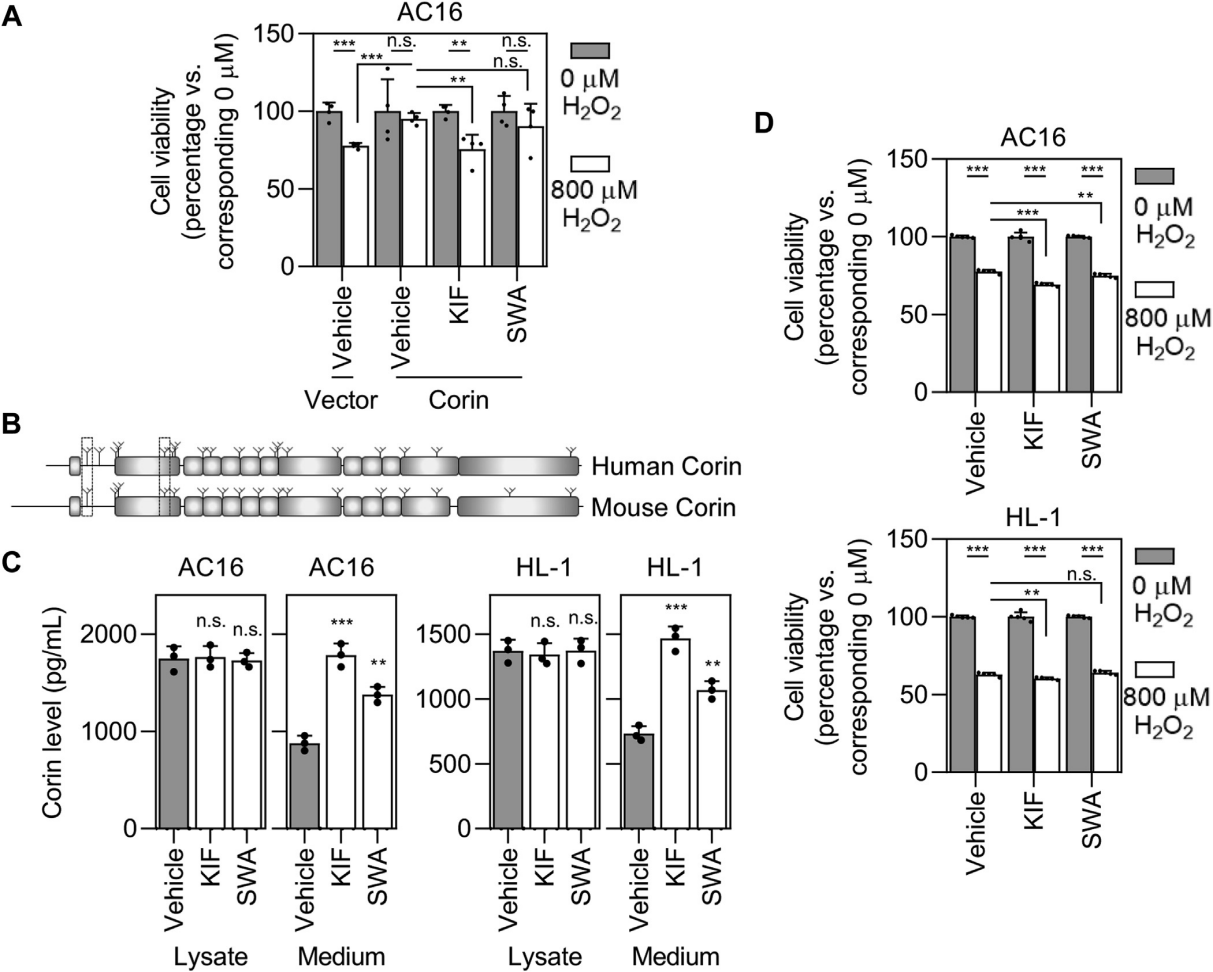
**H**_**2**_**O**_**2**_**-induced injury with inhibition of Golgi α-mannosidases.***A*, cell viability of vector- or corin-expressing AC16 cells with H_2_O_2_ treatment and/or inhibition of Golgi α-mannosidases. Data are means ± SD from four independent experiments. *B*, conservation of ectodomain shedding–related *N*-glycosylation sites in human and mouse corin. The two sites are *circled. C*, corin levels in cell lysate and conditioned medium samples from WT AC16 (*left*) and HL-1 (*right*) cells. Data are means ± SD from three independent experiments. *D*, cell viability of WT AC16 (*top*) and HL-1 (*bottom*) cells with H_2_O_2_ treatment and/or inhibition of Golgi α-mannosidases. Data are means ± SD from five independent experiments. ∗*p* < 0.05; ∗∗*p* < 0.01; and ∗∗∗*p* < 0.001. n.s., not significant.

Corin has been reported to play a protective role in hydrogen peroxide (H_2_O_2_)-induced cardiomyocyte injury (29). To investigate the biological effects of Golgi α-mannosidase–regulated ectodomain shedding on cell survival, we applied H_2_O_2_ treatment to corin-expressing AC16 cells with inhibitors for α-mannosidases. H_2_O_2_ treatment reduced the cell viability of vector-expressing AC16 cells (78 ± 2% of 0 μM for 800 μM, *p* < 0.001; n = 4; the vehicle group), whereas the reduction was not significant in corin-expressing AC16 cells (95 ± 4% of 0 μM for 800 μM, *p* = 0.654; n = 4; the vehicle group), which is consistent with the reported protective role of corin (Fig. 7*A*). Compared with vehicle-treated corin-expressing AC16 cells, cell viability was lower in KIF- and SWA-treated cells with H_2_O_2_ treatment (76 ± 8% of 0 μM for 800 μM in the KIF group, *p* = 0.002; 90 ± 15% of 0 μM for 800 μM in the SWA group, *p* = 0.306; n = 4), indicating that inhibition of Golgi α-mannosidases could aggravate H_2_O_2_-induced injury in AC16 cells by regulating corin expression and function (Fig. 7*A*).

The two *N*-glycosylation sites, that regulate corin ectodomain shedding, are conserved among corin proteins of different species, including human and mouse (Figs. 7*B* and S4). The endogenous corin proteins from human and murine cardiomyocytes (WT AC16 and HL-1 cells, respectively) were analyzed with inhibitors of α-mannosidases. Increased ectodomain shedding was also detected (1785 ± 120 pg/ml with KIF, 1378 ± 80 pg/ml with SWA, *versus* 878 ± 76 pg/ml with the vehicle in AC16 cells; 1466 ± 91 pg/ml with KIF, 1069 ± 68 pg/ml with SWA, *versus* 732 ± 58 pg/ml with the vehicle in HL-1 cells; n = 3; *p* < 0.001, *p* = 0.002, *p* < 0.001, and *p* = 0.003, respectively), further supporting the *N*-glycan–dependent regulation of corin ectodomain shedding by Golgi α-mannosidases (Fig. 7*C*). Similarly, inhibitors of α-mannosidases, especially KIF, sensitized WT AC16 cells to the H_2_O_2_ treatment (69.3 ± 0.9% of 0 μM for 800 μM in the KIF group, 75.0 ± 1.2% of 0 μM for 800 μM in the SWA group, *versus* 77.6 ± 1.2% of 0 μM for 800 μM in the vehicle; n = 5; *p* values < 0.001 and = 0.009) (Fig. 7*D*). Similar results were found in WT HL-1 cells (60.3 ± 0.8% of 0 μM for 800 μM in the KIF group, 64.1 ± 1.2% of 0 μM for 800 μM in the SWA group, *versus* 62.9 ± 1.3% of 0 μM for 800 μM in the vehicle; n = 5; *p* values = 0.004 and 0.156) (Fig. 7*D*).

### Importance of individual α-mannosidases in N-glycan type and corin expression

**Figure 8:**
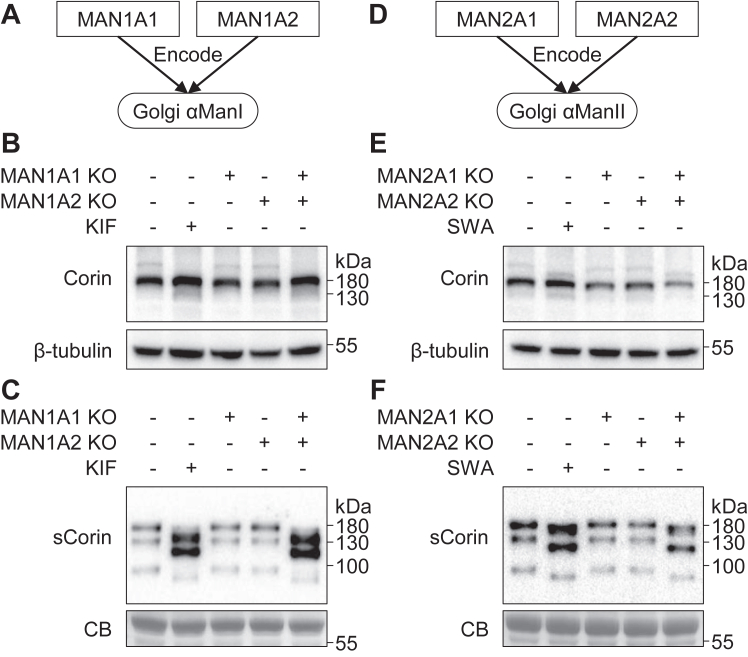
**Gene KO of Golgi α-mannosidases.***A*, genes encoding Golgi α-mannosidase I (αManI). *B* and *C*, Western blot analysis of corin in the cell lysate (*B*) and conditioned medium (*C*) from transfected α-mannosidase I KO HEK293 cells. *D*, genes encoding Golgi α-mannosidase II (αManII). *E*-*F*, Western blot analysis of corin in the cell lysate (*E*) and conditioned medium (*F*) from transfected α-mannosidase II KO HEK293 cells. Two genes encoding the same kind of enzymes were KO (+), individually or together. The corresponding inhibitor-treated (+) cells were included as positive controls. Levels of β-tubulin in the cell lysates and a Coomassie Blue (CB)-stained nonspecific protein in the conditioned medium were used to assess protein amounts in each sample.

In cells, Golgi α-mannosidase I is encoded by *MAN1A1* and *MAN1A2* genes, while Golgi α-mannosidase II is encoded by *MAN2A1* and *MAN2A2* genes (Fig. 8, *A* and *D*) (15, 17). To investigate if either of the paired genes was sufficient to process *N*-glycans on corin, we expressed corin in gene KO HEK293 cells lacking α-mannosidases individually or together. In Western blotting, the migration patterns of corin proteins in cell lysates (Fig. 8*B*) and the conditioned media (Fig. 8*C*) from *MAN1A1* or *MAN1A2* KO cells were similar to those from WT HEK293 cells. Only when both *MAN1A1* and *MAN1A2* were knocked out, did the migration patterns of corin zymogen and soluble fragment bands change and become similar to those in KIF-treated cells (Fig. 8, *B* and *C*). Similar findings were observed in the cells lacking *MAN2A1* and/or *MAN2A2*. In the *MAN2A1/MAN2A2* double KO (*MAN2A* DKO) cells, corin expression levels in the conditioned media were higher than those in the control cells and comparable to those in SWA-treated cells (Fig. 8, *E* and *F*). Endo H digestion also showed that, if only one of the genes was knocked out, the cell surface corin remained resistant to Endo H, an indication of the presence of the complex *N*-glycans (Fig. S5).

### Recovery of zymogen activation by shedding inhibitors

**Figure 9:**
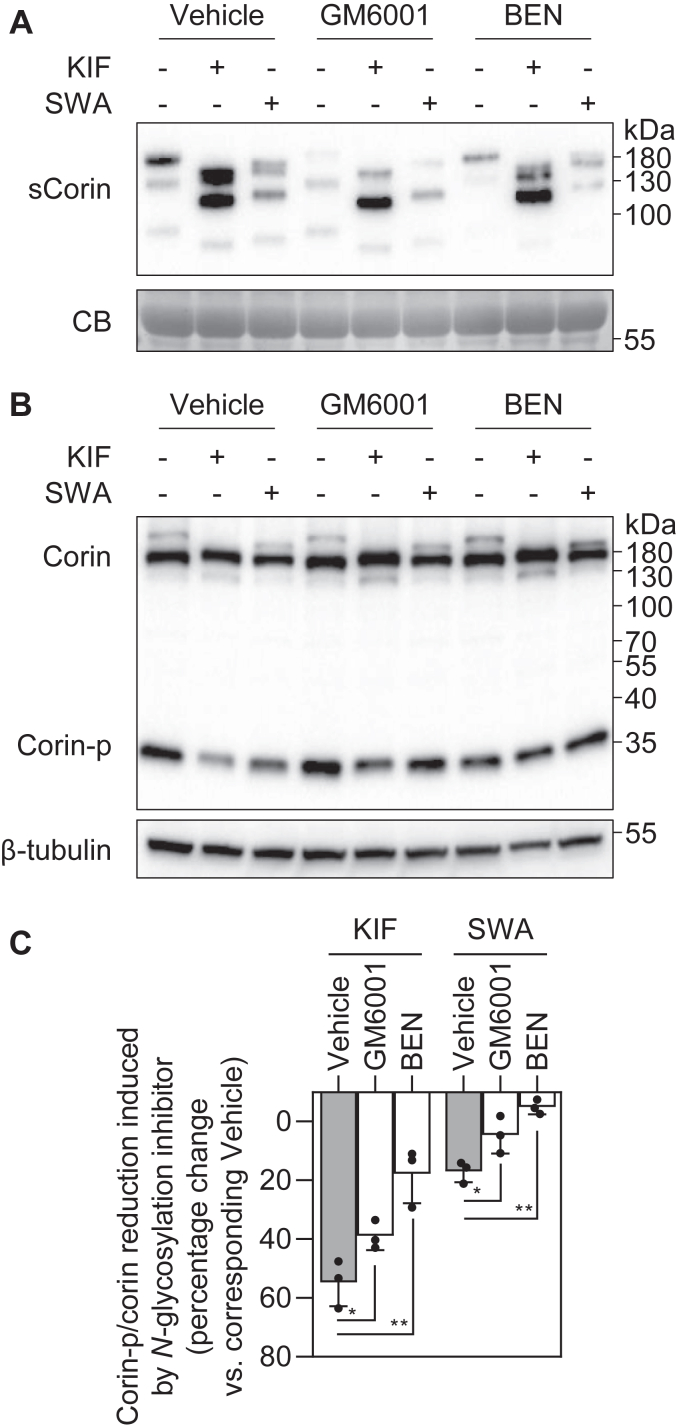
**Inhibition of ectodomain shedding.***A* and *B*, Western blot analysis of corin shedding (*A*) and zymogen activation (*B*) from corin-expressing HEK293 cells treated with (+) or without (−) ectodomain shedding inhibitors and *N*-glycosylation inhibitors. GM6001 inhibited ADAM10-mediated shedding, while BEN inhibited corin autocleavage. Levels of a Coomassie Blue (CB)-stained nonspecific protein in the conditioned medium and β-tubulin in the cell lysates were used to assess protein amounts in each sample. *C*, reduction of the ratio of corin-p *versus* corin bands, as estimated by densitometric analysis of Western blots. The *N*-glycosylation inhibitor–treated samples were compared with corresponding vehicle-treated samples to calculate the reduction. Data are means ± SD from three independent experiments. ∗*p* < 0.05; ∗∗*p* < 0.01 *versus* vehicle. ADAM, a disintegrin and metalloproteinase; BEN, benzamidine.

As shown above, KIF and SWA treatment altered corin ectodomain shedding but not cell surface targeting (Figs. 2 and 3). To examine if inhibition of shedding may restore activated corin on the cell surface, we examined the effects of GM6001 (an inhibitor for ADAM10-mediated corin shedding) and benzamidine (BEN) (an inhibitor for corin autocleavage) on KIF- and SWA-treated HEK293 cells expressing corin. In samples from the conditioned medium, levels of the ADAM10-cleaved top soluble corin fragment were reduced in all the GM6001-treated samples with or without KIF or SWA treatment (Fig. 9*A*). In samples from BEN-treated cells, reduced corin shedding was observed with or without SWA (Fig. 9*A*). In the cells treated with both BEN and KIF, levels of the corin soluble fragments remained high (Fig. 9*A*), suggesting that inhibition of α-mannosidase I may increase the sensitivity of corin autocleavage in the frizzled-1 domain (9). The corin-p bands (indicator of corin activation) were stronger when the cells were treated with either GM6001 or BEN, as shown in Western blotting of cell lysates (Fig. 9*B*). The reduction of ratio of corin-p *versus* zymogen bands induced by KIF decreased in the presence of GM6001 or BEN (39 ± 5 and 18 ± 10%, respectively, *versus* 55 ± 8% with the vehicle; n = 3; *p* values = 0.044 and 0.008) (Fig. 9*C*). Similar decreases were shown in SWA-treated samples in the presence of GM6001 or BEN (4 ± 6 and −5 ± 2%, respectively, *versus* 17 ± 4% with the vehicle; n = 3; *p* values = 0.042 and 0.001) (Fig. 9*C*).

## Discussion

The importance of *N*-glycan processing is widely investigated in glycoproteins. Most of recent studies focus on the effects on *N*-glycome (30, 31). In HepG2 cells, for example, KIF treatment increased Man9 and Man8 *N*-glycans of the high-mannose type in which *N*-glycans were rarely fucosylated. Unlike in KIF treatment, increased *N*-glycans upon SWA treatment were primarily Man5 or the hybrid type, which could undergo fucosylation (30). There have been reports studying how *N*-glycan processing regulates *N*-glycan structures on specific proteins. For example, the recombinant constant fragment of human immunoglobulin G 1 produced in WT HEK293 cells contained only complex *N*-glycans. However, when it was produced in *MAN2A* DKO cells, only hybrid *N*-glycans were detected (15). In the current study, the *N*-glycan type changes on cell surface corin were analyzed by glycosidase digestion and the migration position of corin bands on Western blots. Compared with intracellular corin with Endo H-sensitive nascent *N*-glycans, cell surface corin migrated slower and was Endo H-resistant, indicating that glycosyltransferase transfer, which follows glycosidase trimming, increased the molecular mass of complex *N*-glycans (Figs. 2 and S1). Inhibition of mannosidase trimming by SWA could prevent glycosyltransferase transfer in one branch, which is unfavorable for *N*-glycan–type change from hybrid to complex *N*-glycans and hence molecular mass increase (Fig. 1). Consistently, the cell surface corin band and soluble fragments migrated faster than those in the vehicle-treated samples on Western blots and were sensitive to Endo H (Figs. 2, 3 and S2). When glycosyltransferase transfer in both branches was stopped, corin bands with processed *N*-glycans migrated even faster (Figs. 2, 3 and S2). These results indicate that inhibition of α-mannosidase I or II could alter the structures and molecular masses of corin *N*-glycans on the cell surface.

The effects of glycosidases on cell surface *N*-glycan types rely on their subcellular locations along the *N*-glycan processing pathway (15, 17, 32). Glucosidase trimming is a key step in calnexin/calreticulin-assisted protein folding (18, 33, 34). The glucosidase inhibitor, 1-DNJ, treatment impairs the expression of cell surface corin (18). In this study, we found that 1-DNJ treatment did not alter the migration pattern or Endo H sensitivity of corin (Figs. 2, 3 and S2). Our results are consistent with previous findings that α-mannosidases could remove mannose residues in *N*-glycans with the linked glucose residues (19). Both α-mannosidases I and II, the targets of KIF and SWA, respectively, are present in the ER and Golgi (15, 17, 30). As illustrated in Figure 1, corin is synthesized and *N*-glycosylated in the ER and travels to the Golgi and cell surface. Golgi α-mannosidases I and II serve as gatekeepers in the conversions from high-mannose to hybrid and from hybrid to complex *N*-glycans. In *MAN1A1/MAN1A2* DKO (*MAN1A* DKO) HEK293 cells without Golgi α-mannosidase I, the migration pattern and Endo H sensitivity of corin proteins were similar to those in the KIF-treated WT HEK293 cells (Figs. 8 and S5). Similar results were observed between *MAN2A* DKO HEK293 cells and SWA-treated WT HEK293 cells (Figs. 8 and S5). Moreover, the results in single *MAN1A* (*MAN1A1* or *MAN1A2*) or *MAN2A* (*MAN2A1* or *MAN2A2*) KO cells showed that either single gene encoding α-mannosidase I or II is sufficient for processing *N*-glycans on corin, resulting in complex *N*-glycans on cell surface corin (Figs. 8 and S5). These results indicate the importance and effectiveness of Golgi α-mannosidases I and II in the *N*-glycan–type conversion of corin.

The glucosidase trimming plays a local role in the ER. Monoglucosylated *N*-glycans, the intermediate generated by glucosidase trimming, directly interact with the ER chaperone, calnexin, to assist corin protein folding (18). In our study, however, inhibition of α-mannosidase trimming in the Golgi increased ectodomain shedding of corin, which occurs on cell surface, remote from the functioning location of α-mannosidases. These results indicate that inhibition of α-mannosidase activities did not block corin intracellular trafficking in the Golgi but enhanced ectodomain shedding on the cell surface. Previously, we found that *N*-glycans at Asn-80 and Asn-231 in corin could inhibit the shedding of ∼962-AA (∼180 kDa) and 878-AA (∼160 kDa) soluble fragments, respectively (12). Possibly, *N*-glycans at Asn-80 and Asn-231 block the access of the shedding enzymes to their cleavage sites (12). In the current study, levels of the ∼962-AA and 878-AA fragments increased when α-mannosidases were inhibited or deleted. These results indicate that *N*-glycans modified by Golgi α-mannosidases are likely to regulate corin ectodomain shedding at Asn-80 and Asn-231. As shown in Western blotting, α-mannosidase inhibition reduced molecular mass of corin, indicating that corin proteins from the inhibitor-treated cells contained *N*-glycans with fewer monosaccharide residues and hence smaller spatial sizes. Compared with high-mannose/hybrid *N*-glycans, complex *N*-glycans have more galactoses and sialic acids but fewer mannoses. It is possible that inhibition of Golgi α-mannosidases alters *N*-glycan structures and reduces their sizes, making it more accessible for the shedding enzymes to cleave Asn-80 and Asn-231 in corin on the cell surface. This hypothesis was also supported by analyzing the 3D models of corin domains with different types of *N*-glycans (Figs. 6 and S3).

Several genetic or pathological factors have been reported to regulate the function of corin. For example, corin variants with reduced zymogen activation and pro-ANP processing activity have been identified in hypertensive patients (5, 10, 11, 35, 36, 37). Recently, a gene association analysis also reported eight loss-of-function (nonsense, frameshift, and splice-site) *CORIN* mutations in a cohort of patients with myocardial infraction (38). Additionally, a microRNA, miR-1-3p, was found to inhibit corin activity in human induced pluripotent stem cell–derived cardiomyocytes *via* binding to the 3′ UTR of *CORIN* (39). Inositol-requiring protein 1 triggered by ER stress in advanced heart failure could cause corin mRNA delay and protein deficiency in cardiomyocytes (40). Our previous studies have shown that *N*-glycosylation at specific sites is a critical regulator of corin activities (12, 18). To date, few genetic variants or mutations at *N*-glycosylation sites of corin have been reported (5, 10, 11, 35, 36, 37, 38). In general, the genetic disorder and drug-induced dysfunction in *N*-glycan processing are known to alter *N*-glycoprotein activities, contributing to diseases in humans (16, 20, 21, 22, 23, 24, 25, 27, 41, 42, 43, 44, 45, 46). The results in this study suggest that altering *N*-glycan processing, either by genetic defects or drug treatment, could be potential mechanisms in corin dysfunction, which may lead to cardiac injury under oxidative stress or hypertension (Figs. 5 and 7). Moreover, our results also suggest that the inhibition of ectodomain shedding may be a therapeutic strategy to enhance corin activity in patients with defects in natriuretic peptide processing.

## Experimental procedures

### Cell culture

HEK293 (American Type Culture Collection, CRL-1573, short tandem repeat profiled), AC16 (MeisenCTCC, CTCC-003-0014, short tandem repeat profiled), and HL-1 (short tandem repeat profiled) cells were grown in Dulbecco’s Modified Eagle’s medium (Gibco, C11995500BT) with 10% fetal bovine serum (Gibco, 16000044) and 100 U/ml penicillin-streptomycin (Gibco, 15140122) at 37 °C in humidified incubators with 5% CO_2_ and 95% air.

### Generation of corin-stable cells

The plasmid expressing recombinant corin WT with a C-terminal V5 tag was described previously (47). The lentivirus expressing the corin protein was generated by GeneChem Corporation, carrying the protein-coding sequence from corin WT and a puromycin-resistance gene. HEK293 cells were transfected with the corin plasmid using FuGENE reagents (Promega, E231A), according to the manufacturer’s instructions. AC16 cells were transfected by incubation with the corin WT lentivirus. Plasmid-transfected HEK293 cells or virus-transfected AC16 cells were cultured with G418 (400 μg/ml for HEK293 cells, Gibco, 10131035) or puromycin (2 μg/ml for AC16 cells, Beyotime, ST551). After ∼2 week, G418-resistant HEK293 cells or puromycin-resistant AC16 cells were selected and stable corin expression was verified by Western blotting.

### Inhibition of N-glycosylation and/or ectodomain shedding

Cells expressing corin were incubated with *N*-glycosylation inhibitors and/or ectodomain shedding inhibitors at 37 °C for 48 h. *N*-glycosylation inhibitors used in this study included NGI-1 (10 μM, MedChemExpress, HY-117383), 1-DNJ (2 mM, MedChemExpress, HY-14860), KIF (10 μM, MedChemExpress, HY-19332), and SWA (50 μM, Yuanye Bio-Technology, B20684). Ectodomain shedding inhibitors included Ilomastat (GM6001, 50 μM, MedChemExpress, HY-15768) and BEN (5 mM, MedChemExpress, HY-W018781). The cells incubated with vehicle (dimethyl sulfoxide and/or buffer) were used as controls. Protein expression in the conditioned medium and cell lysates was analyzed in the following analyses.

### Western blotting of recombinant proteins

Recombinant corin fragments in the conditioned medium were immunoprecipitated by an anti-V5 antibody (1:2000, Invitrogen, R960-25). Cells were lysed in NP-40 lysis buffer (Beyotime, P0013F) containing a protease inhibitor mixture (Beyotime, P1050). Proteins in the lysates were analyzed by Western blotting under reducing conditions in the presence of DTT (Beyotime, P0015L). To examine corin shedding, protein samples from the conditioned medium were analyzed by Western blotting under nonreducing conditions in the absence of DTT (Bio-Rad, 1610737). The protein samples were separated by SDS-PAGE and corin proteins were analyzed using a horseradish peroxidase–conjugated anti-V5 antibody (1:5000, Invitrogen, R961-25). β-tubulin in cell lysates was examined using an anti-β-tubulin primary antibody (1:1000, Affinity, T0023) and a horseradish peroxidase–conjugated secondary antibody (1:1000, Beyotime, A0216). Western blots were analyzed using an imaging system (Bio-Rad, Universal Hood III), and the protein bands representing corin fragments were quantified by the Image Lab software (Bio-Rad, https://www.bio-rad.com/zh-cn/product/image-lab-software?ID=KRE6P5E8Z).

### Glycosidase digestion

The protein samples, denatured as described above, were incubated with a reaction buffer containing Endo H (New England BioLabs, P0702S) or PNGase F (Yeasen, 20411ES01), according to the manufacturers’ instructions. After 2 h at 37 °C, the glycosidase-treated corin proteins were analyzed by SDS-PAGE and Western blotting, as described above.

### Flow cytometry

Inhibitor-treated corin-stable HEK293 cells were detached by flushing with PBS (KeyGen BioTECH, KGB5001) and incubated with an anti-V5 primary antibody (1:200, Invitrogen, R960-25), followed with an Alexa Fluor 488-conjugated secondary antibody (1:200, Cell Signaling Technology, 4408S). Life-cell gating was performed with pyridinium iodide (BD Biosciences, 51-66211E). The stained cells were filtered with the cell strainer (Falcon, 352350) to remove cell aggregates. Data were collected on a flow cytometer (Sony, MA900) and analyzed by FlowJo software (https://www.flowjo.com/).

### Pro-ANP processing

The conditioned medium containing recombinant human pro-ANP was collected from stable HEK293 cells expressing human pro-ANP and incubated with inhibitor-treated corin-stable HEK293 cells at 37 °C for 30 min. Pro-ANP and ANP in the conditioned medium were analyzed by immunoprecipitation and Western blotting (12, 35).

### Simulation of 3D models of N-glycopeptides

The corin amino acid sequence (residues 67–263), covering juxtamembrane and frizzled-1 domains, were submitted to a fully automated server (SWISS-MODEL) and a 3D model of the peptide were generated based on an AlpahFold database model of human corin (48, 49, 50). Glycoprotein Builder, a web-tool offered by GLYCAM-Web server (http://glycam.org), simulated the 3D *N*-glycopeptide models using the 3D peptide model generated by SWISS-MODEL server and the 3D *N*-glycan models from 3D Structure Libraries (for glycans) on GLYCAM-Web server. The 3D *N*-glycopeptide models were inspected and imaged with the PyMOL package (Schrodinger, LLC. 2010. The PyMOL Molecular Graphics System, version 2.3.0., https://pymol.org/2/).

### Cell viability analysis of H_2_O_2_-treated cardiomyocytes

The vector-expressing AC16 cells were generated in the same way as corin-expressing AC16 cells. Cardiomyocytes, including vector-expressing, corin-expressing, or WT AC16 cells and WT HL-1 cells were cultured with 800 μM of H_2_O_2_ for 24 h to induce cell injury. *N*-glycosylation inhibitors and vehicle were added together with H_2_O_2_ to investigate the effects of Golgi α-mannosidase–regulated ectodomain shedding. After incubation, cell viability was assessed by a cell counting kit-8 (Beyotime, C0039), following the manufacturer’s instructions. The absorbance at 450 nm was measured using a Sunrise microplate reader (TECAN).

### ELISA for endogenous corin in cardiomyocytes

The lysates and media of WT AC16 and HL-1 cells were prepared as described above. The protein concentration of lysates was measured. According to the protein concentration, the lysates were further diluted with lysis buffer to make final protein concentrations of each lysate sample equal. The corin levels in the lysate and medium samples were quantified by an ELISA (R&D Systems, DY2209).

### Corin expression in gene KO cells

Genes encoding α-mannosidases I and II were knocked out in HEK293 cells using the CRISPR/Cas9 system, as described previously (15, 17). The gene KO cells included *MAN1A1* KO, *MAN1A2* KO, *MAN1A* DKO, *MAN2A1* KO, *MAN2A2* KO, and *MAN2A* DKO cells. The cells were transfected with the corin plasmid, and corin proteins were analyzed 48 to 60 h after transfection, as described above.

### Statistical analysis

Analysis was performed with Prism 8 software (Graphpad, https://www.graphpad.com/). Normal distribution of the data was confirmed using Shapiro-Wilk test. The student’s *t* test was used to compare data from two groups, and the one-way ANOVA test was used to compare data from three or more groups. A *p* value of <0.05 was considered to be statistically significant.

## Data availability

All data are contained within the article and the supporting information.

## Supporting information

This article contains supporting information.

## Conflict of interest

The authors declare that they have no conflicts of interest with the contents of this article.
