# Supplementary material for: Golgi α-mannosidases regulate cell surface N-glycan type and ectodomain shedding of the transmembrane protease corin
Source: J Biol Chem. 2023 Sep 1;299(10):105211. doi: 10.1016/j.jbc.2023.105211 (PMC10520876; doi:10.1016/j.jbc.2023.105211)
Supplement: Supplemental data [file mmc1.pdf]

Figure S1 **Corin biosynthesis and the corresponding fragments on Western blots**
(Supplemental Figure)

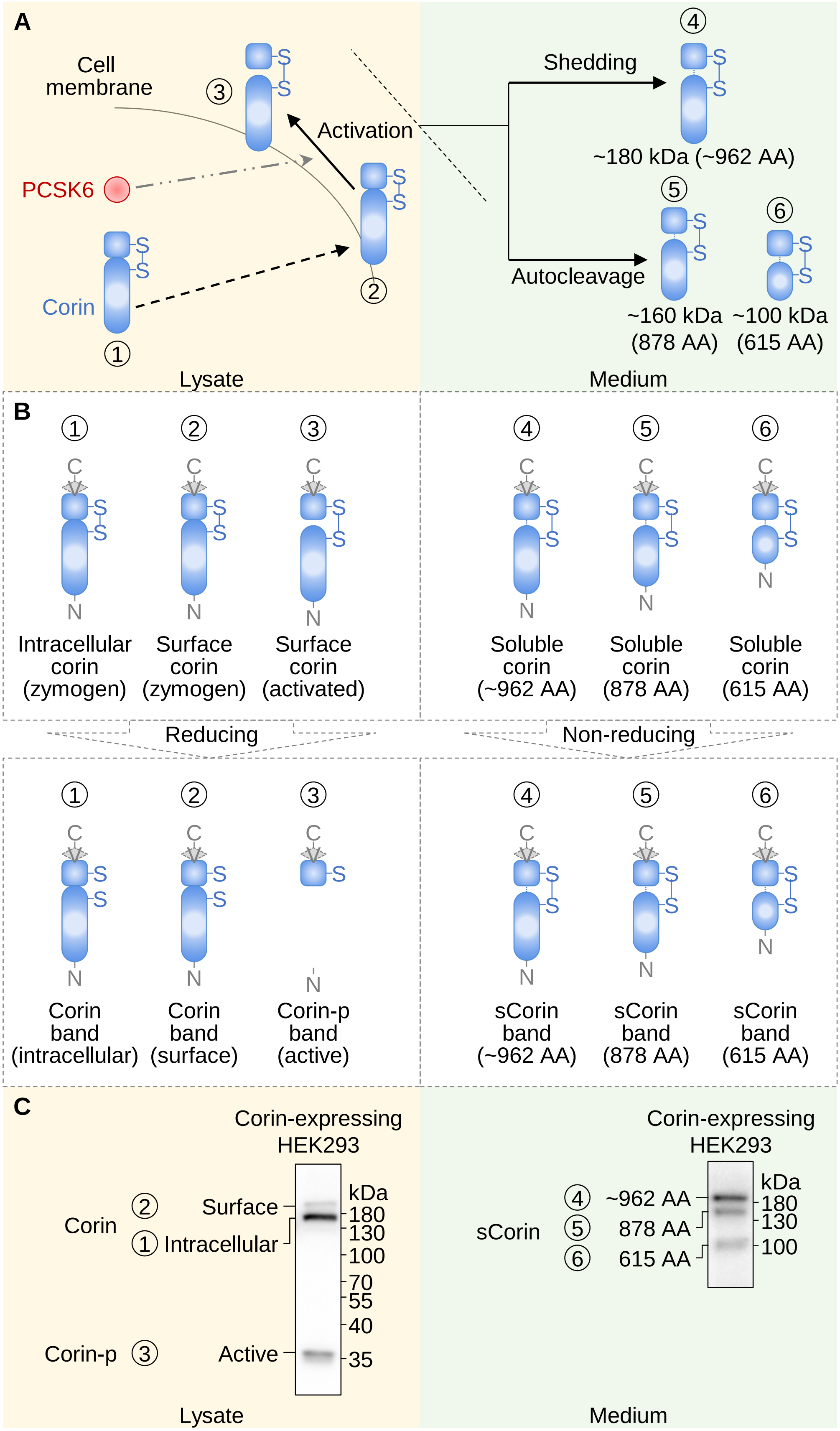

Figure S1. Corin biosynthesis and the corresponding fragments on Western blots. *A,* corin biosynthesis. Since proprotein convertase subtilisin/kexin type 6 (PCSK6), the activator of corin, is secreted in an intracellular trafficking pathway distinct from corin, the activated corin only exists (3) on cell surface. *B,* sample preparation for Western blot analysis of corin proteins. The reducing reagent breaks the disulfide bond between protease domain and pro-domain to release the activated protease domain fragment. The N-terminal, C-terminal and V5-tag were labeled in grey. *C,* Western blots of corin proteins in lysate and medium. Corin bands were detected by an anti-V5 antibody. Band 1 represents intracellular corin zymogen, Band 2 represents surface corin zymogen, Band 3 represents surface activated corin, Band 4 represents ~962-AA soluble corin fragment, Band 5 represents 878-AA soluble corin fragment and Band 6 represents 615-AA soluble corin fragment.

Figure S2 ***N*-glycan types on corin in AC16 cardiomyocytes treated with inhibitors of *N*-glycosylation (Supplemental Figure)**

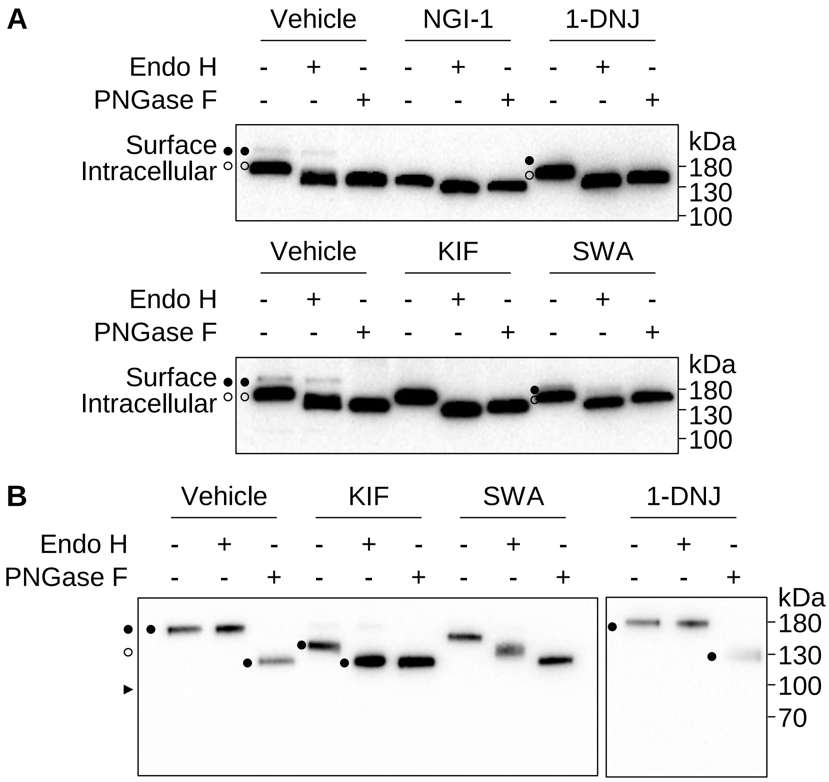

Figure S2. *N*-glycan types on corin in AC16 cardiomyocytes treated with inhibitors of *N*-glycosylation. *A*, Western blot analysis of corin in cell lysates with (+) Endo H digestion, PNGase F digestion or without (-) glycosidase digestion. The cell surface (black dots) and intracellular (white dots) bands are indicated. *B*, Western blot analysis of soluble corin in conditioned medium with (+) Endo H digestion, PNGase F digestion or without (-) glycosidase digestion. The bands of the ~962-AA (black dots), 878-AA (white dots) and 615-AA (black arrowheads) soluble fragments are indicated.

Figure S3 **Surface models of corin juxtamembrane domains with different types of *N*-glycans (Supplemental Figure)**

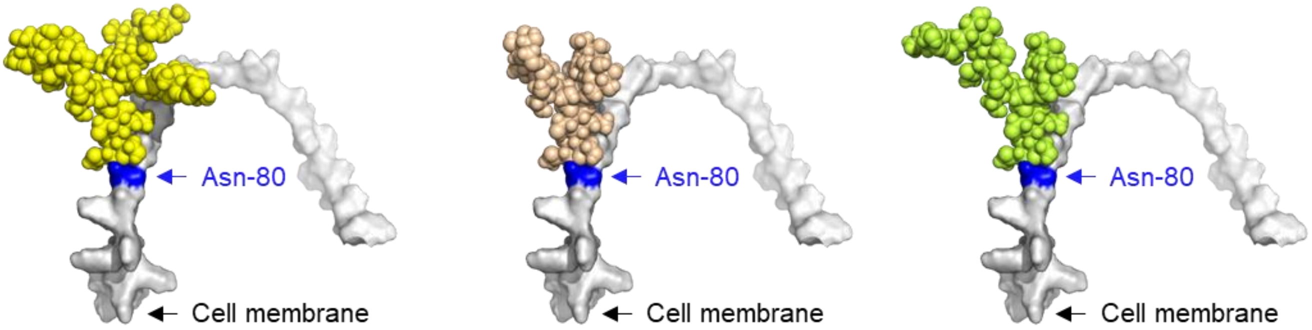

Figure S3. Surface models of corin juxtamembrane domains with different types of *N*-glycans . Complex (yellow), high-mannose (wheat) and hybrid (limon) *N*-glycans are displayed in sphere mode. The *N*-glycosylation (Asn-80, blue) site and the location of cell membrane are indicated.

Figure S4 **Conservation of ectodomain shedding-related *N*-glycosylation sites among corin proteins of different species (Supplemental Figure)**

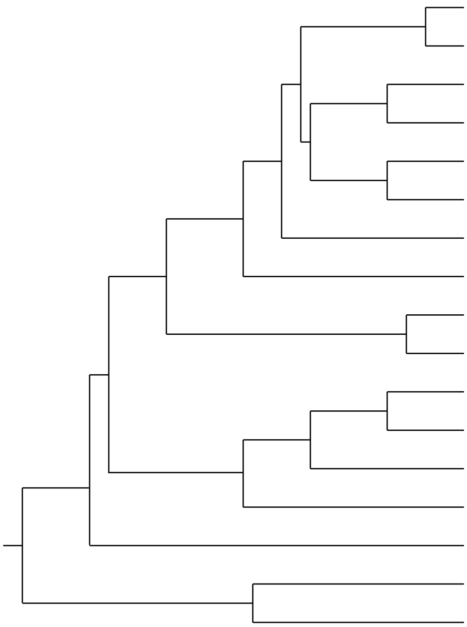

Species	Identity	Ectodomain shedding -related *N*-glycosylation Site		NCBI Reference Sequence
Homo Sapiens	100%	80	231	NP_006578.2
Macaca Fascicularis	96%	80	231	XP_005554886.1
Felis Catus	89%	83	234	XP_019685110.1
Equus Asinus	89%	83	234	XP_014712620.1
Bos Taurus	87%	83	234	XP_005207990.1
Sus Scrofa	87%	83	234	XP_020956233.1
Myotis Brandtii	86%	83	234	XP_005853201.1
Oryctolagus Cuniculus	87%	78	227	XP_008246803.1
Rattus Norvegicus	83%	147	296	NP_872279.1
Mus Musculus	83%	147	298	NP_058565.2
Gallus Gallus	81%	84	320	XP_015140976.1
Corvus Cornix Cornix	71%	51	287	XP_019142590.2
Alligator Mississippiensis	76%	86	320	XP_019336747.1
Pogona Vitticeps	69%	130	349	XP_020645321.1
Xenopus Tropicalis	66%	83	309	XP_004911266.1
Danio Rerio	63%	78	225	XP_021325728.1
Fundulus Heteroclitus	58%	100	262	XP_012713428.2

Figure S4. Conservation of ectodomain shedding-related *N*-glycosylation sites among corin proteins of different species . The phylogenetic relationships of corin in different species were evaluated using the COBALT server at the National Center for Biotechnology Information (NCBI) website based on the full-length corin amino acid sequences. The identity (vs. Homo sapiens), the locations of conserved ectodomain shedding-related *N*-glycosylation sites, and the NCBI references are shown.

Figure S5 ***N*-glycan types on corin in transfected Golgi α-mannosidase KO HEK293 cells (Supplemental Figure)**

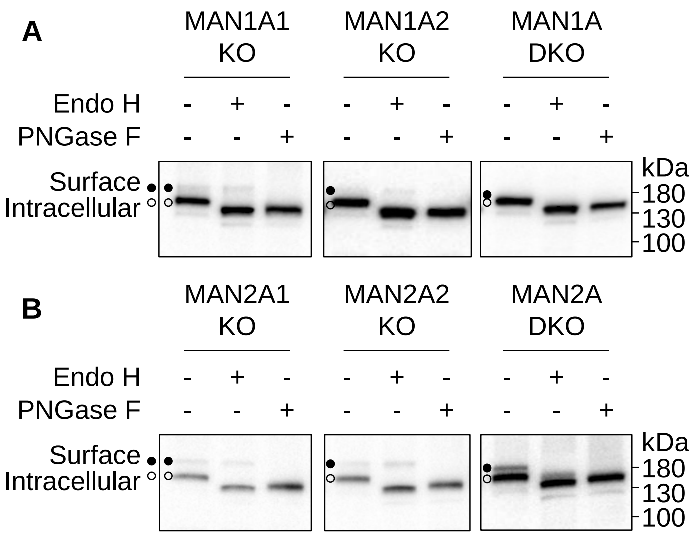

Figure S5. *N*-glycan types on corin in transfected Golgi α-mannosidase KO HEK293 cells. *A*, Western blot analysis of corin in cell lysates from transfected α-mannosidase I KO HEK293 cells with (+) Endo H digestion, PNGase F digestion or without (-) glycosidase digestion. *B*, Western blot analysis of corin in cell lysates from transfected α-mannosidase II KO HEK293 cells with (+) Endo H digestion, PNGase F digestion or without (-) glycosidase digestion. The cell surface (black dots) and intracellular (white dots) bands are indicated. DKO, double knockout.
